# Role-specific patterns of work-related musculoskeletal disorders across nursing settings: a comparative study of operating room, intensive care unit, and ward nurses

**DOI:** 10.3389/fpubh.2026.1813358

**Published:** 2026-06-16

**Authors:** Dichao Zheng, Hui Yu

**Affiliations:** Yuyao People's Hospital of Zhejiang Province, Yuyao, Zhengjiang, China

**Keywords:** cross-sectional studies, ergonomics, intensive care units, musculoskeletal disorders, nurses, operating room

## Abstract

**Background:**

Nurses are at high risk for work-related musculoskeletal disorders. While previous studies often focus on single populations, comparative data across different specialties, including Operating Room, Intensive Care Unit, and General Ward, are limited. This study aimed to compare the prevalence patterns of work-related musculoskeletal disorders across these three distinct nursing roles.

**Methods:**

A cross-sectional study was conducted with 128 nurses (43 operating room nurses, 42 intensive care unit nurses, and 43 ward nurses). Data were collected using the Extended Nordic Musculoskeletal Questionnaire and a demographic/occupational questionnaire. Differences in prevalence across groups were analyzed using Chi-square tests. Multivariate logistic regression was performed to identify role-specific risks for anatomical regions with a total positive case count exceeding 45 to ensure model stability.

**Results:**

Significant differences in work-related musculoskeletal disorders prevalence were observed across the three groups. Operating room nurses had the highest prevalence of neck (74.4%) and shoulder (69.8%) disorders. Ward nurses exhibited the highest prevalence of lower back (74.4%) and knee (51.2%) disorders. Intensive care unit nurses showed a mixed pattern, intermediate between the other two groups. Multivariate logistic regression confirmed these role-specific risks. Compared to ward nurses, operating room nurses had significantly higher odds of neck work-related musculoskeletal disorders (OR = 4.60, 95% CI: 1.70–13.40, *p* = 0.003) and shoulder work-related musculoskeletal disorders (OR = 3.77, 95% CI: 1.50–10.01, *p* = 0.006). Conversely, operating room nurses had significantly lower odds of knee work-related musculoskeletal disorders compared to ward nurses (OR = 0.25, 95% CI: 0.09–0.65, *p* = 0.006).

**Conclusions:**

Different nursing roles are associated with distinct work-related musculoskeletal disorders patterns, reflecting their unique ergonomic exposures. Operating room nurses are predominantly at risk for upper quadrant disorders (neck/shoulders), while ward nurses face higher risks in the lower body (knees/lower back). These findings underscore the need for role-specific ergonomic interventions and preventive strategies in nursing practice.

## 1 Introduction

Work-related musculoskeletal disorders (WMSDs) represent one of the most significant occupational health burdens globally, affecting millions of workers across various industries. WMSDs encompass a range of painful and debilitating conditions affecting muscles, tendons, nerves, and joints, resulting from cumulative exposure to ergonomic risk factors such as awkward postures, repetitive movements, and heavy lifting (1). The healthcare sector, particularly nursing, is severely affected by the WMSDs (2).

The epidemiological burden of WMSDs among nurses is substantial. A recent meta-analysis by Wang et al. (3) encompassing 23 studies and over 21,000 nurses reported a pooled lifetime prevalence of WMSDs of 79%, with the most commonly affected sites being the neck (58%), waist (57%), shoulders (49%), and back (35%). A cross-sectional study focusing on European nurses found a 12-month prevalence of WMSDs approximately 70% in overall prevalence, with lower back and neck being the leading painful body areas (4). These figures are not merely statistical; they translate into significant personal suffering, reduced quality of life, and substantial economic costs related to sick leave, reduced productivity, and high staff turnover (5).

While the high prevalence of WMSDs in nursing is well-established, the specific patterns of injury across different nursing specialties are less understood. The majority of existing research has focused on single nursing populations. For example, studies have documented high rates of neck and shoulder pain among operating room (OR) nurses, attributing these to prolonged static postures during surgery (6). Research on intensive care unit (ICU) nurses has highlighted the physical demands of frequent patient repositioning and equipment handling, leading to a combination of lower back and shoulder complaints (7). Similarly, studies on general nurses have consistently identified lower back and knee pain as predominant issues, related to extensive walking, bending, and patient transfers (8, 9). While these individual studies are valuable, they do not provide a comparative framework to understand how injury profiles differ systematically between these distinct roles.

The operating room (OR) environment, for instance, presents unique ergonomic challenges including prolonged standing, fixed postures, and restricted movement due to sterile fields and surgical equipment (10). In contrast, intensive care unit (ICU) nurses engage in frequent bedside procedures, patient repositioning, and equipment handling (11), while general ward nurses perform extensive walking, and patient transfers. These distinct work characteristics likely result in different patterns of musculoskeletal loading and, consequently, different WMSDs profiles. Yet, systematic comparisons across these three major nursing roles, OR, ICU, and general ward, remains scarce. Understanding these role-specific patterns is essential for developing targeted preventive interventions. For example, interventions for OR environment might include position changes, regular microbreaks, and adjusting environment (12), whereas interventions for general nursing might prioritize the hospital safety culture, safe patient handling and mobility, and education/training (13).

Therefore, this study aimed to: (1) compare the prevalence and anatomical distribution of WMSDs among OR nurses, ICU nurses, and general ward nurses; and (2) examine the associations between work scenarios and WMSDs across the structures of their bodies. The findings are intended to inform the development of role-specific, evidence-based ergonomic interventions, and occupational health policies.

## 2 Methods

### 2.1 Study design and participants

This study was conducted as a cross-sectional study. The study was reported according to the STROBE (Strengthening the Reporting of Observational Studies in Epidemiology) guidelines to ensure comprehensive and transparent reporting (14). Nurses were recruited from three distinct clinical settings including Operating Rooms (OR), Intensive Care Units (ICU), and General Medical/Surgical Wards at a tertiary hospital. The inclusion criteria were: (1) currently working as a registered nurse in the respective unit; (2) at least 1 year of experience in the current specialty; and (3) willingness to provide informed consent. Exclusion criteria included: (1) work interruption exceeding 3 months in the past year (e.g., maternity leave, long-term sick leave); (2) history of congenital musculoskeletal disorders or spinal/limb surgery; and (3) current pregnancy, as pregnancy independently contributes to multiple musculoskeletal conditions (e.g., low back pain, carpal tunnel syndrome) that could confound the association between occupational exposure and WMSDs.

The sample size was calculated based on an expected prevalence of 50% for WMSDs, with a 95% confidence level and 80% power. To detect significant differences between the three groups, a minimum of 105 participants was required. To enhance representativeness and minimize potential selection bias, we aimed to recruit at least 85% of all eligible nurses in each department. This resulted in a final sample of 128 nurses with 43 participants in the OR group, 42 in the ICU group, and 43 in the ward group, exceeding the minimum calculated requirement.

Recruitment was conducted during routine staff meetings in each participating unit. An investigator provided detailed information about the study purpose, procedures, and confidentiality. Interested nurses who met eligibility criteria were invited to provide written informed consent. Participants were informed of their right to withdraw at any time without consequence.

This study was approved by the Institutional Review Board of the participating hospital. All procedures were performed in accordance with the Declaration of Helsinki. Written informed consent was obtained from all participants before data collection. Participants were informed of their right to withdraw at any time without consequence. All data were de-identified and stored securely, accessible only to authorized research personnel.

### 2.2 Data collection instruments

Data were collected using a structured questionnaire comprising two sections:

1. Demographic and Work Characteristics Form: This form collected information on age, sex, total years of nursing experience, years in current unit, work shift type (day, rotating, or night shifts), and regular exercise participation.
2. Extended Nordic Musculoskeletal Questionnaire (NMQ-E): This validated tool assesses musculoskeletal symptoms in nine anatomical regions (neck, shoulders, upper back, elbows, wrists/hands, lower back, hips/thighs, knees, and ankles/feet) over the past 12 months and 7 days (15). For each region, participants reported whether they had experienced pain, discomfort, or reduced function in the past 12 months and the past 7 days. Additional questions addressed healthcare seeking, medication use, work absenteeism, and reduced daily activities due to these symptoms. The NMQ-E has demonstrated good reliability (Cronbach's α ≥ 0.87) (16).

### 2.3 Data collection procedure

Questionnaires were distributed to enrolled participants during the staff meetings or immediately after informed consent. Participants were given the option to complete the questionnaire on-site or return it within 3 days. Anonymity was ensured by assigning unique participant codes; no personally identifiable information (e.g., name, employee ID) was collected. Upon return, questionnaires were checked for completeness by a research assistant. Incomplete questionnaires were returned to the participant for completion when possible.

### 2.4 Data management and quality control

All data were entered into a Microsoft Excel spreadsheet by two independent researchers (double data entry) to minimize transcription errors. After entry, the two datasets were compared for consistency; discrepancies were resolved by checking the original questionnaires. The final dataset was then imported into IBM SPSS Statistics for Windows, Version 26.0 (IBM Corp., Armonk, NY, USA) for statistical analysis. Data analysis was conducted by an investigator who was not involved in the data collection process to reduce potential bias.

### 2.5 Statistical analysis

Data were analyzed using SPSS version 26.0. Descriptive statistics were presented as frequencies and percentages for categorical variables and means with standard deviations (SD) for continuous variables. Group comparisons were performed using one-way ANOVA for normally distributed continuous data and Chi-square tests for categorical data (prevalence rates). Normality was assessed using the Shapiro-Wilk test.

To identify the association between nursing role and specific WMSDs, multivariate logistic regression models were constructed. Only anatomical regions with a total positive case count exceeding 45 were included to ensure model stability. Model fit was assessed using the Hosmer-Lemeshow goodness-of-fit test, and the events-per-variable (EPV) ratio was calculated to ensure model stability. Odds ratios (OR) with 95% confidence intervals (CI) were calculated, with Ward nurses serving as the reference group. Statistical significance was set at *p* < 0.05.

## 3 Results

### 3.1 Participant characteristics

A total of 128 nurses participated in the study, with 43 in the OR group, 42 in the ICU group, and 43 in the ward group. The mean age of participants was 32.3 years (SD = 6.3), and 82.0% were female. The mean total nursing experience was 9.0 years (SD = 5.9), and mean experience in the current unit was 5.9 years (SD = 3.7). Regarding work shifts, 62.5% worked rotating shifts, 25.8% worked day shifts only, and 11.7% worked night shifts only. Regular exercise was reported by 68.0% of participants. No significant differences were observed in baseline characteristics across the three groups (Table 1). No participants had missing data for any of the demographic or occupational variables.

**Table 1:** Participant characteristics by nursing role.

Characteristic	Total (*N* = 128)	OR Nurses (*n* = 43)	ICU Nurses (*n* = 42)	Ward Nurses (*n* = 43)	*p-value*
Age (years), mean ± SD	32.3 ± 6.3	32.0 ± 6.3	32.4 ± 6.9	32.5 ± 6.0	0.926
Sex (female), *n* (%)	105 (82.0)	35(81.4)	35 (83.3)	35 (81.4)	0.965
Total working years, mean ± SD	9.0 ± 5.9	9.1 ± 5.7	8.7 ± 5.5	9.3 ± 6.4	0.900
Years in current unit, mean ± SD	5.9 ± 3.7	5.4 ± 3.6	6.1 ± 3.6	6.1 ± 3.8	0.556
Work shift, *n* (%)					0.413
Day shift only	33 (25.8)	11 (25.6)	11 (26.2)	11 (25.6)	
Rotating shifts	80 (62.5)	27 (62.8)	26 (61.9)	27 (62.8)	
Night shift only	15 (11.7)	5 (11.6)	5 (11.9)	5 (11.6)	
Regular exercise, *n* (%)	87 (68.0)	30 (69.8)	29 (69.0)	28 (65.1)	0.884

OR, operating room; ICU, intensive care unit; SD, standard deviation.

### 3.2 Prevalence of WMSDs by anatomical region

The 12-month prevalence of WMSDs across all participants was highest for the lower back (68.8%, 88/128), followed by the neck (59.4%, 76/128), shoulders (54.7%, 70/128), and upper back (42.2%, 54/128). The lowest prevalences were observed for the elbows (18.8%, 24/128) and hips/thighs (28.1%, 36/128). Detailed prevalence rates by anatomical region are presented in Table 2.

**Table 2:** Twelve-month prevalence of WMSDs by anatomical region and nursing role.

Anatomical region	Total (*N* = 128) *n* (%)	OR nurses (*n* = 43) *n* (%)	ICU nurses (*n* = 42) *n* (%)	Ward nurses (*n* = 43) *n* (%)	*p-value*
Neck	76 (59.4)	32 (74.4)	24 (57.1)	20 (46.5)	0.029
Shoulders	70 (54.7)	30 (69.8)	22 (52.4)	18 (41.9)	0.032
Upper back	54 (42.2)	18 (41.9)	18 (42.9)	18 (41.9)	0.994
Elbows	24 (18.8)	8 (18.6)	8 (19.0)	8 (18.6)	0.998
Wrists/hands	42 (32.8)	14 (32.6)	14 (33.3)	14 (32.6)	0.996
Lower back	88 (68.8)	26 (60.5)	30 (71.4)	32 (74.4)	0.34
Hips/thighs	36 (28.1)	12 (27.9)	12 (28.6)	12 (27.9)	0.997
Knees	46 (35.9)	10 (23.3)	14 (33.3)	22 (51.2)	0.024
Ankles/feet	42 (32.8)	14 (32.6)	14 (33.3)	14 (32.6)	0.996

WMSDs, work-related musculoskeletal disorders; OR, operating room; ICU, intensive care unit.

### 3.3 Comparison of WMSDs across nursing roles

Significant differences in WMSDs prevalence were observed across the three groups for several anatomical regions (Table 2). OR nurses demonstrated the highest prevalence of neck disorders (74.4%, 32/43), followed by ICU nurses (57.1%, 24/42) and ward nurses (46.5%, 20/43) (χ^2^ = 7.09, *p* = 0.029). Similarly, shoulder disorders were most prevalent among OR nurses (69.8%, 30/43), compared to ICU nurses (52.4%, 22/42) and ward nurses (41.9%, 18/43) (χ^2^ = 6.91, *p* = 0.032).

In contrast, ward nurses exhibited the highest prevalence of lower back disorders (74.4%, 32/43), followed by ICU nurses (71.4%, 30/42) and OR nurses (60.5%, 26/43), although this difference did not reach statistical significance (χ^2^ = 2.16, *p* = 0.34). Knee disorders were significantly more common in ward nurses (51.2%, 22/43) than in ICU nurses (33.3%, 14/42) or OR nurses (23.3%, 10/43) (χ^2^ = 7.52, *p* = 0.024).

ICU nurses showed a mixed pattern, with intermediate prevalence rates for most regions. Notably, they had the highest prevalence of upper back disorders (42.9%, 18/42), though this was not statistically significant (χ^2^ = 0.01, *p* = 0.994).

### 3.4 Work-related consequences of WMSDs

Among participants reporting WMSDs, lower back symptoms were the most common reason for healthcare seeking (22.7%, 29/128), medication use (20.3%, 26/128), and sick leave (22.5%, 27/128). Functional impairment in daily activities was most frequently attributed to lower back (38.3%, 49/128), followed by neck (29.7%, 38/128) and shoulders (25.0%, 32/128). Detailed consequences by anatomical region are presented in Table 3. Participants could report multiple regions; percentages are based on the total sample of 128.

**Table 3:** Work-related consequences of WMSDs by anatomical region (*N* = 128).

Anatomical region	Healthcare seeking *n* (%)	Medication use *n* (%)	Sick leave *n* (%)	Functional impairment *n* (%)
Neck	27 (21.1)	23 (18.0)	18 (15.0)	38 (29.7)
Shoulders	24 (18.8)	20 (15.6)	16 (13.3)	32 (25.0)
Upper back	18 (14.1)	15 (11.7)	10 (8.3)	23 (18.0)
Elbows	6 (4.7)	5 (3.9)	2 (1.7)	9 (7.0)
Wrists/hands	11 (8.6)	9 (7.0)	6 (5.0)	15 (11.7)
Lower back	29 (22.7)	26 (20.3)	27 (22.5)	49 (38.3)
Hips/thighs	9 (7.0)	7 (5.5)	5 (4.2)	13 (10.2)
Knees	15 (11.7)	12 (9.4)	10 (8.3)	22 (17.2)
Ankles/feet	12 (9.4)	10 (7.8)	7 (5.8)	32 (25.0)

WMSDs, work-related musculoskeletal disorders. Participants could report multiple regions; percentages are based on total sample of 128 nurses.

### 3.5 Factors associated with WMSDs

Multivariate logistic regression analyses were performed for anatomical regions with total positive case counts exceeding 45, specifically the neck, shoulders, upper back, lower back, and knees. The events-per-variable (EPV) ratio for these models ranged from approximately 7.7 (knees) to 14.7 (lower back), with the number of independent variables limited to a maximum of 6 per model to ensure stability. Model fit was assessed using the Hosmer-Lemeshow goodness-of-fit test, with all *p-values* > 0.05, indicating acceptable fit.

The results confirmed the role-specific risks observed in the univariate analysis (Table 4). Compared to ward nurses, OR nurses had significantly higher odds of neck WMSDs (OR = 4.60, 95% CI: 1.70–13.40, *p* = 0.003) and shoulder WMSDs (OR = 3.77, 95% CI: 1.50–10.01, *p* = 0.006). Conversely, OR nurses had significantly lower odds of knee WMSDs compared to ward nurses (OR = 0.25, 95% CI: 0.09–0.65, *p* = 0.006). No significant associations were observed between nursing role and lower back or upper back WMSDs in the multivariate models.

**Table 4:** Multivariate logistic regression analysis of factors associated with WMSDs.

Region	Group (vs. Ward)	OR	95% CI	*p-value*
Knees	ICU	0.41	0.15–1.04	0.064
	OR	0.25	0.09–0.65	0.006
Lower_back	ICU	0.87	0.32–2.33	0.783
	OR	0.54	0.20–1.36	0.194
Neck	ICU	1.57	0.62–4.06	0.343
	OR	4.60	1.70–13.40	0.003
Shoulders	ICU	1.56	0.64–3.87	0.335
	OR	3.77	1.50–10.01	0.006
Upper_back	ICU	1.04	0.43–2.51	0.926
	OR	1.01	0.42–2.41	0.989

WMSDs, work-related musculoskeletal disorders; OR, odds ratio; CI, confidence interval; ref, reference category; OR nurses, operating room nurses; ICU nurses, intensive care unit nurses. Only regions with total positive cases exceeding 45 were included to ensure model stability. All models were adjusted for age and sex.

## 4 Discussion

To our knowledge, this study provides the first systematic comparison of WMSDs patterns across three major nursing roles: OR nurses, ICU nurses, and general ward nurses. Our findings indicate that different nursing roles are associated with distinct profiles of musculoskeletal disorders, reflecting the unique ergonomic exposures inherent to each work environment.

### 4.1 Role-specific WMSDs patterns

The high prevalence of neck and shoulder disorders among OR nurses aligns with the findings of Ocaktan and Karabacak (6), who reported neck and shoulder as the most affected sites in this population. This pattern likely reflects the static, prolonged postures required during surgery, particularly when using microscopes or endoscopic equipment that necessitate sustained neck flexion or rotation (17). The sterile field restricts movement, preventing nurses from changing positions or taking micro-breaks, leading to cumulative muscle fatigue and strain.

In contrast, ward nurses exhibited the highest prevalence of lower back and knee disorders. This finding is consistent with the dynamic nature of ward work, which involves frequent walking, bending, lifting, and transferring patients. These activities impose repetitive mechanical loads on the lumbar spine and lower extremities, increasing the risk of degenerative changes over time (18). The higher knee disorder prevalence among ward nurses may also reflect prolonged standing combined with frequent stooping and kneeling during patient care (19).

ICU nurses demonstrated an intermediate pattern, with significant burdens in both upper and lower body regions. This mixed profile likely reflects the hybrid nature of ICU work, which combines prolonged standing (similar to OR nurses) with frequent patient handling and equipment manipulation (similar to ward nurses). The high prevalence of upper back disorders in this group may be associated with the need to maintain awkward postures while performing procedures at the bedside or managing ventilators and monitors positioned at suboptimal heights (20).

### 4.2 Protective factors

While regular exercise emerged as a protective factor, it is unlikely to be the sole modifiable factor influencing WMSDs risk. A growing body of literature has identified several other protective factors that warrant consideration. First, ergonomic training and education have been shown to significantly reduce WMSDs incidence when combined with equipment provision. A systematic review by Santos and colleagues supports ergonomic strategies as an effective practical approach to reduce WMSDs (21). Second, availability and use of assistive devices such as ceiling lifts, sliding sheets, and adjustable height beds have consistently been associated with lower rates of handling injuries (22). Third, organizational factors, including adequate staffing levels, supportive supervision, and a positive safety climate, have been shown to buffer the physical demands of nursing work (23). Finally, psychosocial factors such as job control, social support from colleagues, and recognition from management have been associated with reduced musculoskeletal symptom reporting. A systematic review and meta-analysis by Bernal and colleagues found that high levels of social support were associated with a lower risk of MSDs, suggesting that a supportive work environment may buffer the impact of physical demands (24). Our findings, combined with this broader evidence, suggest that a comprehensive, multi-level approach to prevention, targeting individual behaviors, workplace equipment, and organizational culture, is likely to be most effective.

### 4.3 Clinical and educational implications

Our findings have several implications for clinical practice and nursing administration. At our institution, current ergonomic provisions for OR nurses include anti-fatigue mats and adjustable stools, yet these have not been systematically evaluated. Our data showing high neck and shoulder symptom prevalence in OR nurses suggest that these measures are insufficient. Based on our findings, we recommend supplementing existing equipment with scheduled micro-breaks (e.g., 5-min breaks every 90 min during long procedures) and targeted ergonomic training focused on neck-sparing postures during microscope use. For ward nurses, where lower back and knee symptoms predominate, current safe patient handling equipment (including ceiling lifts and sliding sheets) is available but underutilized. Our findings support intensified training on the use of this equipment and consideration of knee protection strategies, such as padded kneeling mats, for tasks requiring sustained kneeling positions. For ICU nurses, a hybrid approach combining both sets of strategies is warranted.

### 4.5 Strengths and limitations

This study has several strengths, including the systematic comparison across three distinct nursing roles, the use of a validated instrument (NMQ-E), and the examination of multiple individual and work-related factors. However, several limitations should be acknowledged.

First, the cross-sectional design precludes causal inferences. While we identified associations between nursing roles and WMSDs, we cannot determine whether these relationships are causal or reflect other unmeasured confounding factors. Second, the singularity of data structures may limit generalizability to other settings with different patient populations, equipment, or work organization. We have therefore emphasized that our findings pertain to this specific context. Third, WMSDs were assessed through self-report, which is subject to recall bias. However, the NMQ-E has been widely used and demonstrates good reliability for assessing musculoskeletal symptoms. Fourth, the relatively small sample size (approximately 43 per group) may have limited our ability to detect smaller differences between groups. The events-per-variable ratio for some models (e.g., knees) was below the recommended threshold of 10, and these findings should be interpreted with caution. Fifth, we did not conduct direct ergonomic observations using tools such as REBA or RULA, nor did we employ technological assessments such as inertial measurement units (IMUs) or video-based motion analysis. Sixth, our classification of nursing roles assumed relative homogeneity within each group. However, such as scrub nurses and circulating nurses may develop intra-group heterogeneity. Such within-role diversity may dilute or obscure true associations between the nominal role and WMSD outcomes. Future research combining self-report with direct observational methods and wearable sensors would strengthen the evidence base by providing objective measures of postural exposure.

## 5 Conclusions

In conclusion, this study reveals that, in this specific context, operating room, intensive care unit, and ward nurses exhibit distinct patterns of work-related musculoskeletal disorders corresponding to their unique ergonomic exposures. Static postures in OR nurses predispose them to neck and shoulder complaints, while dynamic patient handling in ward nurses elevates lower back and knee risks, with ICU nurses demonstrating a mixed burden. These findings underscore the critical need for role-specific ergonomic interventions and institutional support for nurses' physical wellbeing to mitigate occupational health risks. Larger, multicenter studies incorporating objective exposure assessment are needed to confirm and extend these findings.

## Data Availability

The data are available from the corresponding author upon reasonable request.
